# The Role of Male Reproductive Organs in the Transmission of African Swine Fever—Implications for Transmission

**DOI:** 10.3390/v14010031

**Published:** 2021-12-24

**Authors:** Hanna Roszyk, Kati Franzke, Angele Breithaupt, Paul Deutschmann, Jutta Pikalo, Tessa Carrau, Sandra Blome, Julia Sehl-Ewert

**Affiliations:** 1 Institute of Diagnostic Virology, Friedrich-Loeffler-Institut, Suedufer 10, 17493 Greifswald-Insel Riems, Germany; hanna.roszyk@fli.de (H.R.); Paul.Deutschmann@fli.de (P.D.); jutta.pikalo@gmx.at (J.P.); Tessa.CarrauGarreta@fli.de (T.C.); 2 Institute of Infectology, Friedrich-Loeffler-Institut, Suedufer 10, 17493 Greifswald-Insel Riems, Germany; kati.franzke@fli.de; 3 Department of Experimental Animal Facilities and Biorisk Management, Friedrich-Loeffler-Institut, Suedufer 10, 17493 Greifswald-Insel Riems, Germany; Angele.Breithaupt@fli.de (A.B.); julia.sehl-ewert@fli.de (J.S.-E.)

**Keywords:** African swine fever, male reproductive tract, pathogenesis, virus detection, histopathology, venereal transmission

## Abstract

African swine fever (ASF) has evolved from an exotic animal disease to a threat to global pig production. An important avenue for the wide-spread transmission of animal diseases is their dissemination through boar semen used for artificial insemination. In this context, we investigated the role of male reproductive organs in the transmission of ASF. Mature domestic boars and adolescent wild boars, inoculated with different ASF virus strains, were investigated by means of virological and pathological methods. Additionally, electron microscopy was employed to investigate in vitro inoculated sperm. The viral genome, antigens and the infectious virus could be found in all gonadal tissues and accessory sex glands. The viral antigen and viral mRNAs were mainly found in mononuclear cells of the respective tissues. However, some other cell types, including Leydig, endothelial and stromal cells, were also found positive. Using RNAScope, p72 mRNA could be found in scattered halo cells of the epididymal duct epithelium, which could point to the disruption of the barrier. No direct infection of spermatozoa was observed by immunohistochemistry, or electron microscopy. Taken together, our results strengthen the assumption that ASFV can be transmitted via boar semen. Future studies are needed to explore the excretion dynamics and transmission efficiency.

## 1. Introduction

African swine fever (ASF) is one of the most complex viral diseases affecting livestock and has tremendous socio-economic impact [1]. This impact, and its potential for transboundary spread, has led to its inclusion in the list of notifiable diseases. Over the last 14 years, ASF has evolved from an exotic animal disease to a threat to global pig production and endangered wild suids, now affecting Europe, Asia, Oceania, and first countries in the Americas, in addition to Africa (https://www.oie.int/en/disease/african-swine-fever/, accessed on 15 October 2021).

The roots of ASF lie in sub-Saharan Africa, where ASF virus (ASFV), the sole member of the *Asfarviridae* virus family, is transmitted in an ancient sylvatic cycle among warthogs and the soft ticks of genus *Ornithodoros* [2]. This cycle is not accompanied by overt disease or mortality in warthogs, and would probably go unnoticed. However, any introduction of the disease into the domestic pig sector via ticks or fomites leads to a severe systemic disease that can resemble a viral hemorrhagic fever, with exceptionally high lethality (over 90% of infected animals die). Once the disease has left the sylvatic cycle, the competent arthropod vector is no longer required to sustain infection chains, and the disease is transmitted directly and indirectly among susceptible suids [3].

An important avenue for the wide-spread transmission of animal diseases is the dissemination of viruses through boars and boar semen, as artificial insemination is now practiced on a vast majority of all sows inseminated in countries with industrialized pig production [4]. Thus, the impact of contaminated semen multiplies, with the widespread distribution of semen from centralized boar studs [5].

Little is known regarding the involvement of the male genitals in ASFV infection and its potential transmission via semen. Thacker et al. [5] cite that ASFV was isolated from semen from an experimentally infected viraemic boar, and that transmission occurred to a recipient female. This is in line with our previous study, that demonstrated the viral genome and virus in all relevant gonadal tissues of wild boar infected with a Belgian ASFV strain [6]. Immunohistochemistry of the male gonads revealed ASFV positively labelled cells identified as macrophages, endothelial cells and peritubular fibroblasts based on the cellular phenotype. Inflammatory changes and vasculitis/vasculopathy were observed.

To further elaborate on these data, we took advantage of the opportunity provided by a study to characterize two African ASFV isolates in sexually mature boars. Moreover, we could investigate samples taken from adolescent wild boar in an experiment with a German ASFV strain from Saxony.

We turned to the detectability of ASFV in the reproductive organs and accessory sex glands. In addition to the virological and molecular detection of the virus and its genome, we investigated the distribution of the virus by immunohistochemistry and in situ hybridization.

Furthermore, in vitro inoculation of commercial boar semen was carried out followed by electron microscopic investigations to establish whether ASFV is found in the spermatozoa themselves.

## 2. Materials and Methods

### 2.1. Experimental Design

#### 2.1.1. Trial A

This study part included six seven-month-old intact domestic crossbred boars, kept in the high-containment animal facilities at the Friedrich-Loeffler-Institut (FLI), Greifswald-Insel Riems, Germany. In the animal experiment, all applicable animal welfare regulations including EU Directive 2010/63/EC and institutional guidelines were taken into consideration. The animal experiment was approved by the competent authority (Landesamt für Landwirtschaft, Lebensmittelsicherheit und Fischerei (LALLF) Mecklenburg-Vorpommern) under reference number 7221.3-2-011/19. The choice of virus strains reflected the main purpose of this study, i.e., reference material acquisition and characterization of virus strains.

The individually ear-tagged boars were randomly divided into two separate quarantine pens and received seven days to acclimate upon arrival. Subsequently, each group (n = 3) was inoculated intramuscularly, with approximately 1 × 10^3^ hemadsorbing units 50% (HAU_50_), of either ASFV isolate “KAB 6/2” or “SUM 14/11”, respectively (for details see Section 2.2). Over the study period of eight days, all animals were monitored daily, based on a modified clinical scoring system that assigns severity points to common alterations, e.g., reduced liveliness or feed intake [7]. The clinical assessment was complemented by the recording of rectal body temperature profiles. An elevated temperature was defined as a body temperature ≥40.0 °C. A cumulative clinical score of 15 score points or unjustifiable suffering, as assessed by a veterinarian, were set as humane endpoints. Upon termination of the study, the animals were sacrificed by exsanguination in deep anesthesia with a combination of tiletamine/ zolazepam (Zoletil^®^, Virbac), xylazine (Xylazin 20 mg/mL, medistar, Ascheberg, Germany), and ketamine (Ketamin 100 mg/mL, cp-pharma, Burgdorf, Germany). Subsequently, all animals were subjected to a full necropsy, and macroscopic findings were assessed following the protocol by Galindo-Cardiel [8], with slight modifications [9]. Blood and the following tissues were sampled for subsequent investigations and reference material acquisition: spleen; lung; lymph nodes; liver; kidney; skin; tonsil; testis; epididymis; epididymal sperm; vesicular gland; bulbourethral gland, and prostate. Epididymal sperm was obtained by flushing the ducts of the cauda epididymidis with phosphate-buffered saline (PBS) after dissecting the spermatic cord, according to a modified protocol as previously published [10].

#### 2.1.2. Trial B

Eight adolescent male wild boar, aged roughly five months upon enrolment in the study, were investigated using virological techniques. Given the uniform result, only three representative animals underwent subsequent histopathology and immunohistochemistry. The wild boar originated from the breeding unit at the FLI, and were individually ear-tagged upon transfer to the high-containment facility. The animals were part of the mock-inoculated group in a vaccination/challenge trial. The initial animal experiment was approved by the competent authority (Landesamt für Landwirtschaft, Lebensmittelsicherheit und Fischerei (LALLF) Mecklenburg-Vorpommern) under reference number 7221.3-1-035/21.

After acclimation, the animals were immobilized with tiletamine/ zolazepam (Zoletil^®^, Virbac), administered via blowing pipe, to conduct blood sampling and oro-nasal inoculation with 3 mL of a spleen suspension containing a highly virulent ASFV strain isolated in Germany (Saxony 2020, for details see Section 2.2). Clinical scoring and necropsy were conducted as described above; however, rectal body temperatures were only assessed at the endpoint. Given the younger age of the animals, epididymal sperm could not be obtained.

### 2.2. Viruses

Two Zambian ASFV isolates [11], kindly provided by Dr Christopher Netherton (the Pirbright Institute, Pirbright, UK), were used in the framework of trial A. The ASFV isolate “KAB 6/2” was initially obtained from an Ornithodoros soft tick collected from warthog burrows in Central Kafue National Park, Zambia, which, according to its capsid protein p72 encoding region, belongs to genotype (gt) XI. The ASFV isolate “SUM 14/11” was extracted from a soft tick originating from Sumbu National Park, Zambia, and is considered as gt XIII [12]. Virus stocks were grown and titrated on porcine peripheral monocytic cell (PBMC)-derived macrophages, as previously described [13]. For inoculation purposes, virus suspensions were diluted in phosphate-buffered saline (PBS) to a titer of 10^3^ HAU_50_ per mL. The actual virus doses applied were determined by back titration of the inocula.

The ASFV strain “Germany 2020” was isolated from the spleen of a wild boar piglet found dead in the Federal State of Saxony, District of Goerlitz, municipality Krauschwitz. The virus belongs to gt II (German variant VI, ASFV/GER/2020/WB/IV_SN) and shows very high identity with other epidemic strains in Europe and Asia. The virus was administered at a dose of 10^4^ HAU_50_ per mL.

For in vitro inoculation of sperm, the recombinant and wild boar lung cell-adapted strain ASFV “ArmeniaΔ285LGFPhuCD4” was used, as recently described [14].

### 2.3. Cells

For virus isolation and titration, PBMCs were derived from the EDTA blood of healthy donor pigs and isolated by overlaying on Ficoll-Paque density gradient medium (GE Healthcare Life Sciences, Uppsala, Sweden). The erythrocyte fraction was removed and stored at +4 °C for subsequent use in hemadsorption tests. Following further washing and centrifugation steps of the PBMC fraction, cell count was set to 5 × 10^6^ cells per ml. Cells were suspended in RPMI 1460 medium supplemented with 10 % bovine serum, 0.01% β-mercaptoethanol, penicillin, streptomycin and amphotericin B (Gibco TM Antibiotic-Antimycotic, Thermo Fisher Scientific, Bleiswijk, The Netherlands). Finally, 96-well plates (5 × 10^5^ cells/well) and 24-well plates (2.5 × 10^6^ cells/well) were prepared. After an incubation period at a humidified atmosphere of 5% CO_2_ and 37 °C for 24 h, culture medium was renewed and granulocyte macrophage colony-stimulating factor (GM-CSF) was added at 2 ng/mL. Following another overnight incubation, cells were used for downstream laboratory tests.

### 2.4. Processing of Samples

For the purpose of the presented study, samples of the blood, spleen, testis, epididymis, epididymal semen, vesicular gland, prostate, and bulbourethral gland were processed in triplicates regarding Trial A. In the case of Trial B, equal sampling proceeded while samples were processed uniquely for qPCR and RT-qPCR, and duplicated for virus isolation.

All tissue samples were cut into lentil-size pieces (~100 mg). The pieces were homogenized in 2 mL reaction tubes, each containing a 5 mm metal bead and PBS (1000 µL) using a TissueLyser II (Qiagen) at 30 Hz for 3 min. Epididymal sperm was diluted 1:1 in PBS for usage in the quantitative polymerase chain reaction (qPCR). EDTA blood was aliquoted and, along with homogenates, stored at −20 °C until further use. For further histopathological processing, tissues were fixed in 10% neutral-buffered formalin for at least three weeks.

### 2.5. Pathogen Detection

#### 2.5.1. Quantitative Polymerase Chain Reaction (qPCR)

Nucleic acids were extracted from EDTA blood, diluted epididymal sperm and homogenate supernatants (100 µL per sample) using the NucleoMag^®^ VET kit (Macherey-Nagel, Düren, Germany) on the King Fisher 96 flex platform (Thermo Fischer Scientific) according to the manufacturer’s instructions. To validate the PCR reaction, a universal heterologous internal control DNA [15] was added to all samples and co-extracted. Subsequently, viral nucleic acids (p72 encoding region) were detected following the qPCR protocol published by King et al. [16]. All qPCRs were performed on a Bio-Rad C1000TM thermal cycler with the CFX96TM Real-Time System (Bio-Rad, Hercules, CA, USA).

Results of qPCR were initially recorded as quantification cycle (Cq) values. Using a dilution series of a full ASFV DNA standard, the genome copies in the respective samples were estimated [17].

#### 2.5.2. Reverse Transcription qPCR (RT-qPCR)

With the aim to detect viral p72 mRNA, RT-qPCR was performed following stringent RNA extraction using TRIzol pre-treatment, in combination with the above detailed magnetic bead-based automatic extraction [18]. To eliminate residual DNA, the extracted samples were treated with DNase employing the TURBO DNA-free™ kit (Thermo Scientific) according to the manufacturer’s instructions. Subsequently, RT-qPCR was performed using the QuantiTect^®^ Probe RT-PCR kit (Qiagen, Hilden, Germany) with the primers and probes designed by Tignon et al. [19]. To control for DNA-contamination, each extracted sample also underwent qPCR simultaneously. Positive results in RT-qPCR were only recorded if the qPCR gave a negative result.

#### 2.5.3. Detection of Infectious Virus (Virus Isolation)

To detect the infectious virus in the blood, spleen, male reproductive tissues, and epididymal sperm, virus isolation and hemadsorption tests were carried out according to slightly modified standard procedures [20] on mature PBMC cultures. To this means, EDTA blood and epididymal sperm were diluted 1:10 in PBS. All other samples were used as homogenates, as described above. In a first blind passage, the PBMC cultures were inoculated in duplicate with 200 µL of the respective materials per well, on a 24-well plate. Following an adsorption time of 2 h at 37 °C, in an incubator with a humidified atmosphere (5% CO_2_), cells were gently washed with lukewarm PBS and the medium was renewed. Following incubation (72 h, 37 °C, 5% CO_2_), the plates underwent a freeze/thaw cycle. The resulting culture supernatants were subjected to a routine hemadsorption test, as previously described [21]. Following the same protocols, endpoint virus titrations were carried out for culture supernatants from epididymal sperm.

### 2.6. Histopathology

Formalin-fixed tissue was trimmed, embedded in paraffin wax, and cut at 2–3 µm thick slices. Tissue sections were further processed for the detection of viral antigens by immunohistochemistry (IHC), for the detection of viral mRNA by RNAScope in situ hybridization (ISH), and for pathomorphological analysis.

#### 2.6.1. Immunohistochemistry (IHC) and Semi-Quantitative Scoring of Viral Antigens

Viral antigen detection was performed following the protocol published recently [9], using an in-house rabbit polyclonal primary antibody against the major capsid protein p72 of ASFV. Cells were evaluated positive when showing a fine granular cytoplasmic signal. Histological specimens were analyzed using a Zeiss AXIO Scope A1 microscope with four objectives, allowing magnification between 2.5× and 40×. For immunohistochemical analysis, the area with the highest viral antigen load was selected and scored per high power field, as follows: no antigen (0); 1–3 positive cells (1); 4–15 cells (2); and ≥16 cells (3).

#### 2.6.2. RNAScope In Situ Hybridization (ISH)

In order to detect the replication of the virus beyond immunohistochemistry, RNA in situ hybridization (ISH) was performed on tissues obtained from the domestic boars with RNAScope 2–5 HD Reagent Kit-Red (ACD, Advanced Cell Diagnostics, Newark, CA, USA), according to the manufacturer’s instructions. RNAScope^®^ probes were designed by ACD against p72 mRNA. A positive probe expressing the housekeeping gene peptidylprolyl isomerase B (cyclophilin B, ppib) and a negative bacterial probe expressing dihydrodipicolinate reductase (DapB) were used to verify the sensitive detection of the target mRNA. To eventually compare results after immunohistochemistry and RNAScope in situ hybridization, tissue sections were scored accordingly on a 0–3 scale, as described above.

#### 2.6.3. Dual RNAScope ISH and Immunofluorescence

To identify ASFV-infected cells, double labeling with RNAScope ISH combined with immunofluorescence staining was performed. RNAScope ISH was followed by incubation with a rabbit polyclonal anti-Iba-1 antibody (dilution 1:500, incubation overnight) (FUJIFILM Wako, Madison, WI, USA). Sections were then treated with a goat anti-rabbit secondary antibody Alexa 488 (Invitrogen, Thermo Fisher Scientific) (dilution 1:1000, incubation 1 h). Cellular nuclei were visualized with Hoechst (dilution 1:5000, incubation 15 min) and sections were mounted with Aquatex (MerckKGaA, Darmstadt, Germany).

#### 2.6.4. Pathomorphology and Semi-Quantitative Scoring of Lesions

Pathomorphological changes were assessed on hematoxylin and eosin-stained tissue sections, following a standard protocol as described earlier [9]. Pathohistological changes scored on a 0–3 scale (normal (0); mild (1); moderate (2); severe (3)), are shown in Table 1.

### 2.7. Transmission Electron Microscopy

For transmission electron microscopy (TEM) analysis, 1 × 10^8^ sperm cells suspended in Tyrode’s albumin lactate pyruvate media [22] were inoculated with ASFV “ArmeniaΔ285L-GFPhuCD4” [14], gt II (MOI 5). Sperm cells were incubated with the virus suspension (48 h, 37°C, 5% CO_2_, humidified atmosphere). A negative sperm cell culture control was treated similarly to verify appropriate handling and culture conditions.

Subsequently, inoculated sperm cells were washed three times in 1× PBS by centrifugation at 134× *g*, 10 min, 23 °C. The resulting pellet was treated with fixing solution (2.5% glutaraldehyde buffered in 0.1 M sodium cacodylate (pH 7.2), 300 mosmol, Serva Electrophoresis, Heidelberg, Germany) for at least 2 h at 4 °C, and embedded in 1.8% low-melting agarose (Biozym). Small pieces were postfixed in 1.0% aqueous OsO4, and stained *en bloc* with uranyl acetate. After stepwise dehydration in ethanol, cells were cleared in propylene oxide, infiltrated with Glycid Ether 100 (Serva Electrophoresis), and polymerized at 60 °C for 3 days. 60–70 nm ultrathin sections were prepared with an ultramicrotome (UC7, Leica Microsystems, Wetzlar, Germany) and collected on EM grids (300 mesh, Plano). Finally, the sections were counterstained with uranyl acetate and lead citrate and analyzed with a Tecnai-Spirit TEM (FEI) at an accelerating voltage of 80 kV.

## 3. Results

### 3.1. Clinial Signs and Gross Pathology

#### 3.1.1. Trial A

Upon intramuscular inoculation with 10^3^ HAU_50_ of ASFV strain “KAB 6/2” (gt XI), boars presented with clinical signs and lesions characteristic of severe acute infection with highly virulent ASFV. The clinical signs included pyrexia (>40.0 °C), anorexia, lethargy, respiratory distress, and conjunctivitis. The clinical scores (CS) rapidly increased from day 3 pi to final scores of 7.5 (#61), 16.5 (#51) and 19 points (#48) (see Table S1). Boar #48 suffered from marked pulmonary failure leading to sudden death on day 7 pi. Pig #51 showed swiftly deteriorating general health and was euthanized reaching the humane endpoint. Post-mortem examination revealed accumulation of serosanguinous fluid in abdominal and thoracic cavities of all pigs, focal subcapsular hemorrhages in the kidneys of two pigs, and moderate gall bladder wall edema in a single boar.

In ASFV “SUM 14/11” (gt XIII)-infected animals, non-specific clinical signs were observed starting on day 4 pi, including pyrexia (>40.0 °C), reduced feed intake, lethargy, respiratory distress, conjunctivitis as well as intradermal hemorrhages. On day 8 pi, which has been determined as the end of the experiment, all boars reached a moderate final CS of 6 points (see Table S1).

At necropsy, gross lesions were slightly more pronounced in ASFV-“SUM 14/11”-inoculated pigs than in the ASFV-“KAB 6/2”-inoculated animals, even though the pigs showed milder clinical signs. All ASFV“SUM 14/11”-inoculated boars revealed a mild, diffuse reddening of the scrotum. Serosanguinous ascites were present in all pigs, whereas focal hemorrhages in the kidney only occurred in two animals. In contrast to ASFV “KAB 6/2”, hemorrhages in the lymph nodes were present to a variable extent, mainly affecting the hepatogastric and renal nodes. Gross lung lesions included lack of retraction or focal atelectasis, which was present in two pigs.

#### 3.1.2. Trial B

Eight adolescent wild boar were inoculated oro-nasally with 10^4^ HAU_50_ per ml of ASFV “Germany 2020” (gt II). The animals showed reduced feed intake and lack of liveliness from 4 dpi. Their condition worsened over the next three days, and all animals were sacrificed at 7 dpi. At that point, the animals had a CS of 4 to 6 score points with anorexia, pyrexia (>40.5 °C), slight ataxia, and depression (see Table S2).

Macroscopic lesions included moderate hemorrhages mainly in the renal and hepatogastric lymph nodes, renal petechiae and mild to moderate pulmonary consolidation.

### 3.2. Pathogen Detection

#### 3.2.1. Detection and Quantification of Viral DNA and mRNA

**Figure 1:**
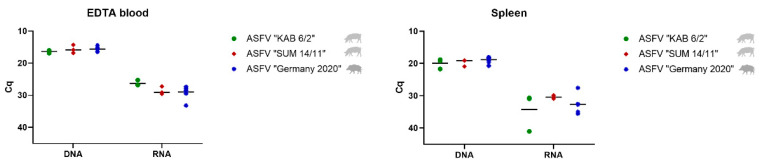
Viral genome loads in EDTA blood and spleen represented by Cq values in qPCR and RT-qPCR. Boars infected with ASFV “KAB 6/2” are depicted by green dots, ASFV-“SUM 14/11”-infected animals by red squares, and ASFV-“Germany 2020”-infected wild boar by blue stars. The mean Cq value per group is represented by a black line.

To detect the ASFV genome in the male reproductive tract of domestic pigs and wild boar, the testis, epididymis and accessory sex glands were analyzed by qPCR and RT-qPCR, and compared to blood and spleen samples (see Figure 1). Table 2 and Table 3 summarize results from qPCR and RT-q PCR (Cq values) from domestic pigs and wild boar, respectively. The corresponding genome copy numbers for ASFV “KAB 6/2” and “SUM 14/11” are shown in Table S3. Copy numbers regarding ASFV “Germany 2020” are depicted in Table S4.

##### Blood and Spleen

In blood samples, the mean Cq values of 16.33 (ASFV “KAB 6/2”), 15.65 (ASFV “SUM 14/11”), and 15.59 (ASFV “Germany 2020”) for viral DNA, and 26.27 (ASFV “KAB 6/2”), 28.62 (ASFV “SUM 14/11”) and 28.96 (ASFV “Germany 2020”) for viral mRNA were detected on days 7 or 8 pi, respectively. In the spleen, viral DNA loads were comparably high in ASFV “KAB 6/2” (mean Cq 19.89) and ASFV “SUM 14/11” (mean Cq 19.68) -infected domestic boars and ASFV “Germany 2020” (mean Cq 18.80) -infected wild boar, whereas viral mRNA amounts were lower with mean Cq values of 34.19 (ASFV “KAB 6/2”), 30.38 (ASFV “SUM 14/11”) and 32.61 (ASFV “Germany 2020”), respectively.

##### Testis and Epididymis

**Figure 2:**
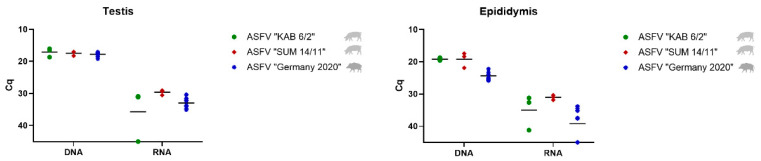
Detection of viral DNA and mRNA in testicular and epididymal samples of mature domestic pigs infected with ASFV “KAB 6/2” (green dots), ASFV “SUM 14/11” (red squares) and prepubescent wild boar infected with ASFV “Germany 2020” (blue stars). The mean Cq value per group is depicted by a black line.

Compared with blood and spleen, viral genome loads were equally high in testicular samples of all animals independent of the ASFV isolate with mean Cq values of 17.03 (ASFV “KAB 6/2”), 17.42 (ASFV “SUM 14/11”), and 17.77 (ASFV “Germany 2020”) (Figure 2). However, viral mRNA loads were lower than the corresponding DNA values with mean Cq values of 35.67 in ASFV “KAB 6/2”- (no detection in animal #48), 29.65 in ASFV “SUM 11/14”- and 32.96 in ASFV “Germany 2020”-infected pigs.

As shown in the testis, high amounts of viral DNA were also detected in the epididymis with mean Cq values of 19.19 for ASFV “KAB 6/2” and 19.23 for ASFV “SUM 14/11”, and a slightly lower value of 24.36 for ASFV “Germany 2020” (Figure 2). When compared to the DNA values, lower values were detected for viral mRNA with mean Cq values of 35.01 (ASFV “KAB 6/2”), 31.04 (ASFV “SUM 14/11”) and 39.18 (ASFV “Germany 2020”).

In epididymal samples of boar #48 (ASFV “KAB 6/2”), as well as in the epididymides of wild boar #13, #15 and #18 (ASFV “Germany 2020”), viral mRNA was not detectable.

##### Prostate, Vesicular and Bulbourethral Gland

**Figure 3:**
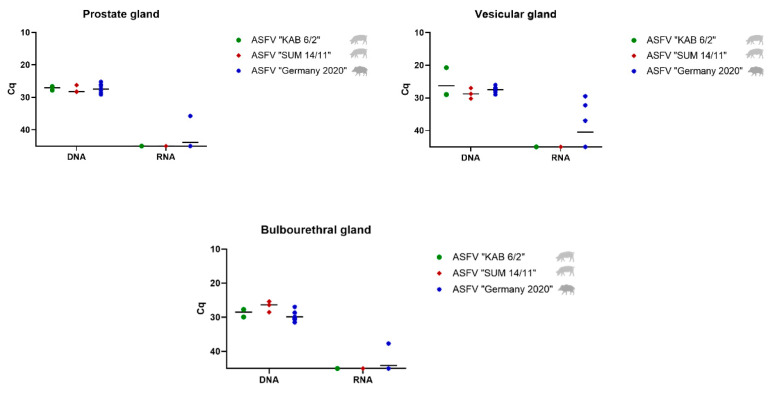
Viral genome and mRNA load in the prostate, vesicular, and bulbourethral glands in ASFV “KAB 6/2”- (green dots) and ASFV “SUM 14/11”-infected domestic pigs (red squares) and ASFV “Germany 2020”-infected wild boar (blue stars). The mean Cq value per group is displayed by a black line.

Unlike the testis and epididymis, only low to moderate amounts of viral DNA were found in the accessory sex glands (Figure 3). More specifically, in prostate samples, mean DNA Cq values reached 27.02 (ASFV “KAB 6/2”), 27.57 (ASFV “SUM 14/11”), and 27.47 (ASFV “Germany 2020”). Similar amounts were present in the bulbourethral gland with mean Cq values of 28.47 (ASFV “KAB 6/2”), 26.73 (ASFV “SUM 14/11”), and 29.81 (ASFV “Germany 2020”), followed by the vesicular gland with 28.87 (ASFV “KAB 6/2”), 28.63 (ASFV “SUM 14/11”), and 27.46 (ASFV “Germany 2020”). While detection of viral mRNA failed in all accessory sex glands of ASFV “KAB 6/2” and “SUM 14/11” infected pigs, it was successful in “Germany 2020”-infected wild boar, with a high mean Cq value of 40.46 for boars #10, #13 and #14 in the vesicular gland, as well as in the prostate gland (wild boar #11) and bulbourethral gland (wild boar #10).

#### 3.2.2. Detection of Infectious Virus

To test for the presence of infectious ASFV, hemadsorption tests were performed on blood, spleen, testis, epididymis, and accessory sex glands (Table 2 and Table 3).

Infectious virus could be isolated from the blood and spleen of all boars, irrespective of the virus isolate used. Except for two pigs infected with “KAB 6/2”, virus isolation from testicular samples succeeded in all animals infected with the three virus isolates.

Virus isolation was also possible from epididymal samples of 2/3 boars of the ASFV “KAB 6/2” group, and of all boars infected with ASFV “SUM 14/11” and ASFV “Germany 2020”.

While infectious virus could be isolated from the vesicular gland of all boars, the prostate gland was positive in all wild boar infected with ASFV “Germany”, but only in 2/3 of those with ASFV “KAB 6/2”, and 2/3 of those with ASFV “SUM 14/11”. Likewise, virus isolation was successful from the bulbourethral gland in 2/3 ASFV “KAB 6/2”-, 2/3 ASFV “SUM 14/11”- and all ASFV “Germany 2020”-infected pigs.

### 3.3. Detection and Quantification of Viral DNA, mRNA and Infectious Virus in Epididymal Sperm

**Figure 4:**
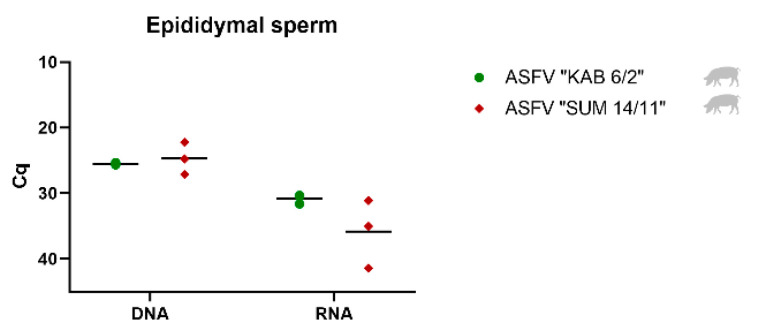
Viral DNA and mRNA loads of epididymal sperm samples of domestic pigs infected with ASFV “KAB 6/2” (green dots) and “SUM 14/11” (red squares).

Epididymal sperm was collected only from mature boars infected with ASFV “KAB 6/2” and “SUM 14/11”, and tested for the presence of viral DNA, mRNA and infectious virus (Figure 4, Table 2). In both groups, moderately high amounts of viral DNA and low amounts of viral mRNA were detected (Figure 4). Viral DNA was found with mean Cq values of 25.48 (ASFV “KAB 6/2”) and 24.69 (ASFV “SUM 14/11”), whereas viral mRNA was present with Cq values of 30.78 (ASFV “KAB 6/2”) and 35.84 (ASFV “SUM 14/11”). All tested epididymal sperm samples were revealed to be infectious by virus isolation (Table 2).

### 3.4. Histopathology

#### 3.4.1. Distribution of Viral Antigen and mRNA in Male Reproductive Organs

**Figure 5:**
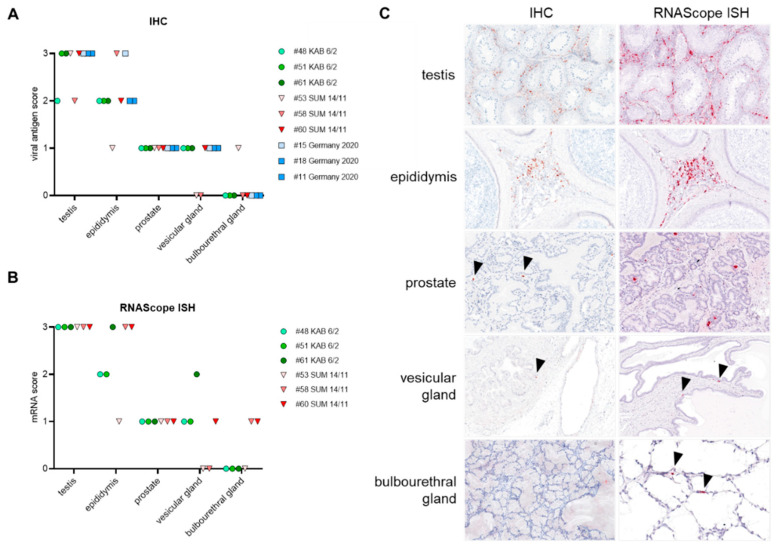
Semiquantitative results of: (**A**) immunohistochemistry (IHC); (**B**) RNAScope in situ hybridization (ISH) of male reproductive organs from ASFV “KAB 6/2”, “SUM 14/11”and “Germany 2020”-infected pigs; (**C**) representative images showing IHC for anti-p72 capsid protein (red-brown signal) and RNAScope ISH (red signal) for anti p72 mRNA of ASFV-infected male reproductive organs. Arrowheads indicate positive cells in less affected accessory sex glands. Tissue sections were counterstained with Mayer’s Hematoxylin.

Tissue specimen of all male reproductive tissues (MRT) were stained against the ASFV main capsid protein p72, using IHC (ASFV “KAB 6/2”, “SUM 14/11”, “Germany 2020”) as well as p72 mRNA using RNAScope ISH (ASFV “KAB 6/2”, “SUM 14/11”), and scored on a 0–3 scale (Figure 5). In the following, the highest IHC scores obtained for each organ of the MRT will be given. As shown by qPCR, the viral antigen amount obtained by IHC was highest in the testis (score 3) and epididymis (score 3). The accessory sex glands showed few positive signals (prostate, score 1; vesicular gland, score 1; bulbourethral gland, score 1), independent of the ASFV isolate used (Figure 5A). Similar results were obtained by RNAScope ISH tested on tissues from ASFV “KAB 6/2” and “SUM 14/11”-infected boar (Figure 5B). However, in some individuals, the frequency of positive signals after RNAScope ISH was higher compared with IHC. Representative sections of the testis, epididymis, prostate, vesicular and the bulbourethral gland after IHC and RNAScope ISH are illustrated in Figure 5C.

#### 3.4.2. Detection of ASFV-Infected Target Cells in the Male Reproductive Tract

**Figure 6:**
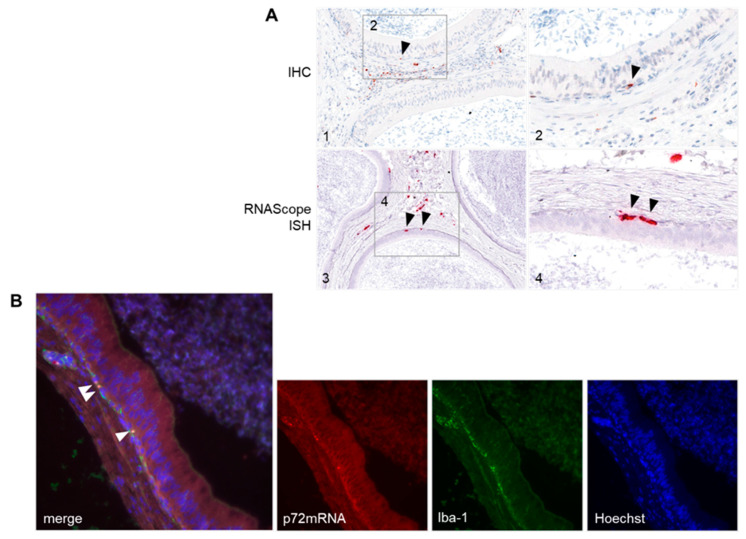
(**A**) Immunohistochemistry (IHC) using a rabbit polyclonal antibody against p72 and RNAScope in situ hybridization (ISH) using a probe against p72 mRNA performed on ASFV-infected epididymis-indicating halo cells (black arrows). Tissues were counterstained with Mayer’s hematoxylin. (**B**) Dual RNAScope in situ hybridization (ISH) and Iba-1 immunofluorescence using a mRNA probe against p72 and a rabbit polyclonal antibody against Iba-1. White arrowheads indicate double staining of ASFV p72mRNA and Iba-1 expression in epididymal halo cells. Tissue sections were counterstained with Hoechst.

In both the testis and epididymis, as well as in the accessory sex glands, IHC identified large mononuclear cells, phenotypically consistent with macrophages, which were predominantly infected by ASFV. To a lesser extent, Leydig cells as well as stromal cells, including smooth muscle cells, fibrocytes, pericytes, and occasionally endothelial cells, were found positive for the p72 antigen in the testis and epididymis, respectively. With IHC against p72 antigen and RNAScope ISH against p72 mRNA, scattered halo cells of the epididymal duct epithelium showed positive signals (Figure 6A). This was confirmed by the double labelling of sporadically Iba-1 positive ASFV-infected cells within the epididymal epithelia using RNAScope ISH against ASFV p72 mRNA, and subsequent immunofluorescence against the macrophage-specific protein Iba-1-identifying halo cells, respectively (Figure 6B).

#### 3.4.3. Histopathological Lesions in the Male Reproductive Tract

Tissue sections of the testis, epididymis, prostate, vesicular and bulbourethral gland obtained from domestic pigs infected with ASFV “KAB 6/2” and “SUM 14/11”, as well as from wild boar infected with “Germany 2020” on day 7 and 8 pi, respectively, were stained with hematoxylin and eosin, and scored for microscopical lesions on a 0–3 scale.

**Figure 7:**
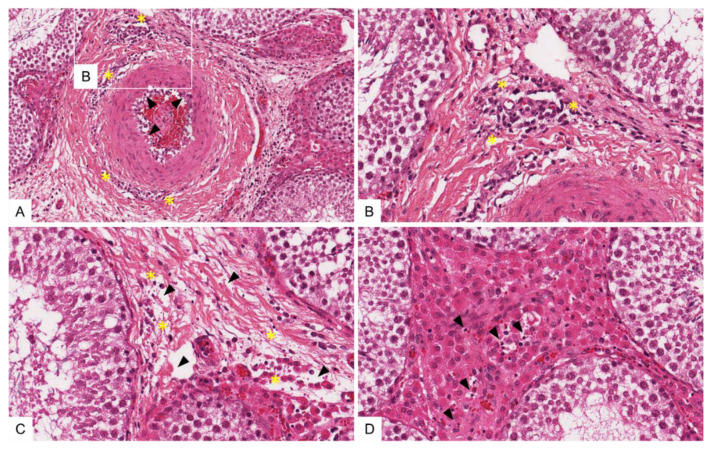
Tissue sections of an ASFV-infected swine testis stained with hematoxylin-eosin. (**A**) Testicular artery showing diffusely activated endothelium (arrow). Vasculitis and perivasculitis of small caliber vessels (rectangle above) expand to the tunica adventitia of the artery (asterisk). (**B**) Magnification of (**A**). Small caliber vessels with activated endothelium, and moderate intramural and perivascular infiltration by mononuclear cells admixed with multiple apoptotic/necrotic cells (asterisk). (**C**) Multifocal single-cell apoptosis/necrosis of cells (asterisk) in mildly edematous testicular stroma (arrows) adjacent to an unaffected seminiferous tubule. (**D**) Focal area showing apoptosis/necrosis of multiple cells (arrows) between normal seminiferous tubules.

**Figure 8:**
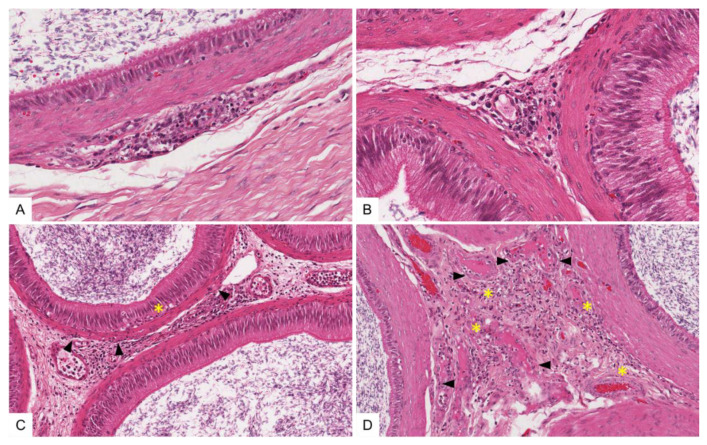
Tissue sections of ASFV-infected swine epididymis, hematoxylin and eosin stain. (**A**,**B**) Moderate necrotizing vasculitis and perivasculitis of a small caliber vessel adjacent to unaffected epididymal ducts. (**C**) Fibromuscular epididymal tissue showing multifocal to coalescing necrotizing inflammation. Smooth muscle cells surrounding epididymal ducts and the basal compartment of the duct epithelium are mildly infiltrated by mononuclear cells (arrows) with occasional single cell apoptosis/necrosis (asterisk). (**D**) Moderate multifocal necrotizing vasculitis affecting small caliber vessels (arrows) and diffuse mononuclear infiltration of fibromuscular tissue admixed with abundant apoptotic/necrotic cells (asterisk).

In general, histopathological changes in the testis and epididymis were slightly more pronounced in ASFV “KAB 6/2”-infected pigs than in the ASFV “SUM 14/11” group. Testicular vessels showed diffuse, prominent endothelial activation and intramural necrotizing inflammation of varying degrees (score 1 to 2) (Figure 7A,B). Ranging from mild to severe, there was extensive apoptosis and/or necrosis of perivascular fibromuscular stromal cells, accompanied by mild lymphohistiocytic infiltration and edema (Figure 7C,D). Likewise, slightly stronger necrotizing vasculitis, perivascular stromal inflammatory infiltration and acute cell loss were detected in the epididymis (Figure 8A–D). However, elongated spermatids were present in both the testis and the epididymis, and none or only minimal infiltration was observed in the seminiferous tubule or epididymal duct epithelia, respectively (Figure 8C). Only minimal changes were detected in the prostate and vesicular gland, comprising occasional single-cell apoptosis/necrosis in the fibromuscular stroma (score 1) and mild, mainly mixed-cellular infiltration (score 1) (data not shown). The bulbourethral gland was unaffected in all animals. Wild boar infected with ASFV “Germany 2020” presented with immature testes, and revealed generally milder lesions compared with “KAB 6/2”- and “SUM 14/11”-infected boar. While all accessory sex glands in wild boar were unaffected, only mild infiltration of the testicular/epididymal fibromuscular stroma with single-cell apoptosis/necrosis, as well as a mild infiltration of single testicular tubules, were observed.

### 3.5. Electron Microscopy of In Vitro Inoculated Sperm

**Figure 9:**
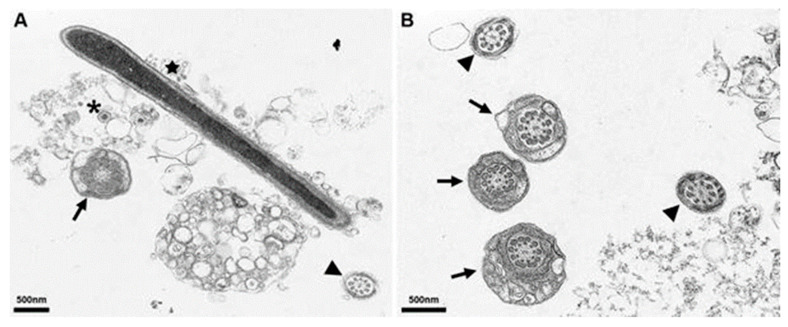
EM Micrographs of sperm from boar. (**A**,**B**) show epoxy resin embedded in vitro inoculated sperm cut in different directions (asterisk). Arrows show the cross section of the head and middle part of a sperm cell, arrowheads show the cross section of the flagellum and the star indicates a longitudinal section of the flagellum. The asterisk represents ASFV particles.

In vitro inoculated sperm was embedded in epoxy resin and cut in different directions. While no virus particles were found inside boar spermatozoa, envelope-free ASFV was detected in the matrix of previously washed sperm cells and inside non-sperm cells. The pattern of distribution appeared to be random without any affinity towards the spermatozoa (see Figure 9).

## 4. Discussion

African swine fever is currently threatening the global pig population with active outbreaks on four continents; thus, understanding disease dynamics and transmission pathways is key when it comes to risk mitigation and prevention. In a globalized world, artificial insemination and international trade of sperm are a widely used instrument for distributing elicited superior genes into sow herds while minimizing the prevalence of transmissible venereal diseases [23]. Yet, once a pathogen passes the high hygiene and precautionary measurements of boar stations, AI threatens to introduce novel pathogens, including transboundary animal diseases such as ASF, into naïve pig populations [24]. The impact that the detection of a notifiable animal disease can have on boar stations was evident in the outbreak of classical swine fever in the Netherlands in 1997/98. There, two semen collection centers became infected. At that time, it was concluded that all the semen collected from the boars at the stations and distributed in a six-week risk period was potentially contaminated. As a consequence, a total of 1680 pig herds were officially declared CSF suspect [25].

Against this background, we undertook further studies to shed light on the possible transmission via boars and boar semen as a high impact avenue for wide-spread dissemination. We took advantage of ongoing animal trials with virulent ASFV strains of different genotypes. In detail, ASFV isolates “KAB 6/2” (gt XI), “SUM 14/11” (gt XIII), and “Germany 2020” (gt II) were used in mature domestic boars and adolescent wild boars, respectively. The trials ended at the height of viraemia, at days 7 or 8 pi. To obtain a broader picture, classical real-time PCRs to detect viral DNA were supplemented with real-time RT-PCRs to detect viral mRNAs. This was done under the assumption that a pure transport of intact virions or any residual DNA could be distinguished from a possible replication. Moreover, we characterized the localization of viral antigen and viral mRNAs using IHC and RNAScope ISH. Finally, we attempted to inoculate commercial boar sperm in vitro, to elaborate on the susceptibility of spermatozoa.

### 4.1. African Swine Fever Virus Is Found in All Male Reproductive Tissues and Epididymal Sperm

In line with our previous studies [6,9], and irrespective of the virus strain or host type involved, ASF viral genome and infectious virus were detected in all organs of the male reproductive tract and in epididymal sperm.

In detail, viral DNA loads were detected in the male reproductive organs, i.e., testis and epididymis, that were *on par* with the loads in the spleen as an organ classically associated with ASF [26]. The detection of viral mRNAs was also accomplished in many cases, pointing at least to replication-competent viruses. Somewhat lower genome loads were found in the still nearly inactive epididymides of the prepubescent wild boar, which may reflect the intensity of blood flow, or the somewhat slower disease progression after oral infection. The results were confirmed by p72 antigen and mRNA detection in IHC and RNAScope ISH, respectively. The accessory sex glands showed only moderate loads of viral genomes, on average 1000-fold lower in the prostate than in the testes, and showed very limited mRNA detections, possibly due to early degradation.

Virus isolation was successful for the majority of domestic pig samples and for all wild boar samples tested. The semen samples also allowed virus isolation. Thus, there is some discrepancy with the detection of viral mRNA. However, it should be noted that virus isolation was performed using an amplifying blind passage. Furthermore, since pure mRNA detection was not a priority, we abstained from measures that would have specifically preserved the integrity of the mRNAs (e.g., chemical stabilization or immediate deep freezing), so that degradation during sample processing was likely. Moreover, one cannot exclude the possibility that infectivity was present in a blood-bound manner, and was therefore not associated with replication directly in the tissues.

The detection of ASFV in the male genitals is in line with the observation in human medicine that not only classical venereal disease pathogens, but also agents causing severe systemic diseases, e.g., Zika or Ebola virus, can be found in the male reproductive tract, including sperm, and can cause persistent infections [27,28]. So far, the putative pathogenesis of these nonclassical viral agents seeding into the male reproductive tract is referred to the testis being an immune-privileged site [29].

### 4.2. Replication Is Linked to Mononuclear Cells of the Respective Tissues

Immunohistochemistry showed that the viral antigens and mRNA were mainly confined to large mononuclear cells, phenotypically consistent with macrophages. Macrophages represent the largest leucocyte populations in testis and epididymis [30,31] and were shown to be the primary target cells of ASFV [32]. Positive signals in both IHC and RNAScope ISH were also detected in epididymal halo cells. Halo cells are considered intraepithelial immune cells of the epididymal duct, but the exact immunological function of these cells is currently still controversial. Several data indicate that halo cells belong to the mononuclear phagocyte system (MPS) [33,34], while others refer to halo cells as T lymphocytes [35]. However, showing high consistency to our results Serre and Robaire [36] showed that halo cells are positive for CD68, a macrophage-specific protein, and recently it was postulated that these cells belong to the MPS, using RNA sequencing [33]. More specifically, halo cells are suspected to contribute to the immunological part of the blood-epididymis barrier [36,37,38,39]. Thus, they can play a crucial role in tissue specific pathogenesis, e.g., by creating proinflammatory environments and facilitating the disruption of barrier integrity.

### 4.3. ASFV Does Not Directly Infect Spermatozoa

Viral antigens were not detected by IHC or RNAScope ISH on the testicular or epididymal germ cell site. However, virus isolation was positive from epididymal sperm. Moreover, the detection of viral mRNA by RT-qPCR indicated the presence of productively infected cells in epididymal sperm. In part, this discrepancy might be explained by blood contamination, cell-free virus particles and/or few migrating infected cells.

In the present experiment, the animals were killed in the viraemic phase, but the disease would probably have taken an even more severe course. Thus, the question remains open as to how the further course of infection affects the distribution of the virus. The detection of viral antigens in halo cells and the signs of inflammation/vasculitis indicated that the integrity of the blood-epithelial barrier may have already been compromised. Especially in presence of systemic inflammation and viremia, the blood-testis/-epididymis/-deferens barriers depicted an insufficient compartmentalization, likely granting access to virions and infected cells [40]. For this reason, the possibility of long-term persistence of ASFV in the male genital tract should also be the subject of future investigations.

With regard to sperm, our EM analyses of in vitro ASFV-inoculated sperm samples points to a low or non-existent susceptibility of mature spermatozoa. However, non-sperm cells were infected, and cell-free virus particles were obvious. Boar sperm always contains seminal plasma and a small fraction of other cells [41], including blood-derived leukocytes that could act as source of infectivity. As entry sites for blood-derived leukocytes, the rete testis and accessory sex glands, particularly the prostate, are suspected [41,42,43]. Consequently, ASFV shedding into sperm might be linked to spermatozoa-accompanying cells and fluids. The same pattern is seen with the Porcine reproductive and respiratory syndrome virus (PRRSV), where round cells were identified as a virus source. Christopher Hennings et al. [34] postulated that PRRSV entered boar semen through replication in reproductive tissue macrophages that became infected upon the viraemia-associated systemic distribution of infected monocytes. Inflammatory processes in the male genitals would most probably lead to higher entry into the sperm.

## 5. Conclusions

Based on our results, it must be assumed that transmission of ASFV via the sexual act or artificial insemination might be possible.

African swine fever in the wild boar population is particularly relevant in Europe. It should also not be ignored that male animals that survive the infection participate in the reproductive process, and that prolonged virus replication in the male reproductive organs, e.g., after initial loss of integrity, may be a problem in maintaining chains of infection.

Since neither the long-term behaviour nor the dose necessary for transmission via sperm are known, only further studies can shed light on these issues.

## Figures and Tables

**Table 1:** List of microscopical criteria evaluated and scored in organs of the male reproductive tract.

	Testis	Epididymis	Prostate	Vesicular Gland	Bulbourethral Gland
Vasculitis/vasculopathy	X	X	X	X	X
Inflammatory infiltration of fibromuscular stroma	X	X	X	X	X
Interstitial single cell apoptosis/necrosis	X	X	X	X	X
Destruction of (tubuloalveolar) glands	-	-	X	X	X
Destruction of seminiferous tubules/epididymal ducts	X	X	-	-	-
Presence/absence of luminal spermatozoa *	X	X	-	-	-

* Findings were only described as present/not present.

**Table 2:** Summarized results of qPCR, RT-qPCR and virus isolation of organ samples of ASFV “KAB 6/2”- and “SUM 14/11”-infected domestic pigs.

Inoculum		ASFV “KAB 6/2”			ASFV “SUM 14/11”		
Animal ID		#48	#51	#61	#53	#58	#60
Age (months)		7			7		
Necropsy (days post inoculation)		7			8		
Final clinical score points		19	16.5	7.5	6	6	6
Blood	DNA Cq value	16.86	16.01	16.13	16.80	15.86	14.28
	mRNA Cq value	25.26	26.75	26.79	29.57	29.08	27.23
	Infectivity	+++	+++	+++	+++	+++	+++
Spleen	DNA Cq value	21.69	18.8	19.18	20.88	19.07	19.08
	mRNA Cq value	41.01	30.91	30.66	30.89	30.39	29.86
	Infectivity	+++	+++	+++	+++	+++	+++
Testis	DNA Cq value	18.65	16.41	16.05	18.25	16.98	17.03
	mRNA Cq value	-	31.14	30.86	30.52	29.02	29.40
	Infectivity	-	-	+++	+++	+++	+++
Epididymis	DNA Cq value	19.55	18.74	19.29	21.90	18.25	17.43
	mRNA Cq value	41.18	31.22	32.62	31.84	30.95	30.34
	Infectivity	-	+++	+++	++	+++	+++
Epididymal sperm	DNA Cq value	25.64	25.49	25.32	22.21	27.12	24.74
	mRNA Cq value	31.59	30.32	30.44	31.10	41.40	35.01
	Infectivity	+++	+++	+++	+++	+++	+++
Prostate gland	DNA Cq value	26.70	27.77	26.6	28.30	28.19	26.21
	mRNA Cq value	-	-	-	-	-	-
	Infectivity	-	+	+++	+++	+	-
Vesicular gland	DNA Cq value	28.90	28.99	28.71	30.19	28.74	26.97
	mRNA Cq value	-	-	-	-	-	-
	Infectivity	+++	+++	+++	+++	+++	+
Bulbourethral gland	DNA Cq value	27.70	29.97	27.74	28.47	26.36	25.35
	mRNA Cq value	-	-	-	-	-	-
	Infectivity	+++	+++	++	++	+++	-

Cq values are given as mean values of three biological replicates of each animal. +++ = tested positive in 3 of 3 replicates; ++ = tested positive in 2 of 3 replicates; + = tested positive in 1 of 3 replicates; - = tested negative.

**Table 3:** Summarized results obtained after qPCR, RT-qPCR and virus isolation of ASFV “Germany 2020”-infected wild boar.

Inoculum		ASFV “Germany 2020”							
Animal ID		#10	#11	#13	#14	#15	#17	#18	#19
Age (months)		6							
Necropsy (days post inoculation)		7							
Final clinical score points		5	4	5	6	5	5	5.5	5
EDTA Blood	DNA Cq value	14.43	15.15	16.41	16.46	15.60	15.50	15.72	15.42
	mRNA Cq value	33.16	28.93	29.43	28.52	27.38	28.93	27.95	27.41
	Infectivity	++	++	++	++	++	++	++	++
Spleen	DNA Cq value	18.10	18.40	18.00	18.38	19.59	18.29	20.70	18.96
	mRNA Cq value	27.54	34.94	32.71	32.50	35.53	32.66	32.43	32.59
	Infectivity	++	++	++	++	++	++	++	++
Testis	DNA Cq value	17.12	17.49	17.07	19.16	17.36	18.50	17.88	17.58
	mRNA Cq value	35.06	34.66	32.13	32.23	30.33	33.71	34.01	31.52
	Infectivity	++	++	++	++	++	++	++	++
Epididymis	DNA Cq value	23.86	23.22	25.53	23.97	22.21	25.27	25.86	24.94
	mRNA Cq value	34.45	37.43	-	35.17	-	37.57	-	33.82
	Infectivity	++	++	++	++	++	++	++	++
Prostate gland	DNA Cq value	26.48	25.17	27.29	27.91	25.96	28.63	29.11	29.18
	mRNA Cq value	-	35.70	-	-	-	-	-	-
	Infectivity	++	++	++	++	++	++	++	++
Vesicular gland	DNA Cq value	27.13	25.99	27.64	28.98	26.82	27.88	26.86	28.37
	mRNA Cq value	36.97	-	29.47	32.23	-	-	-	-
	Infectivity	++	++	++	++	++	++	++	++
Bulbourethral gland	DNA Cq value	30.65	26.92	30.19	31.45	30.27	29.81	30.55	28.63
	mRNA Cq value	37.66	-	-	-	-	-	-	-
	Infectivity	++	++	++	++	++	++	++	++

Cq values are given unique per boar. ++ = tested positive in 2 of 2 replicates; + = tested positive in 1 of 2 replicates.

## Data Availability

The data presented in this study are available on request from the corresponding author.

## Supplementary Materials

The following are available online at https://www.mdpi.com/article/10.3390/v14010031/s1, Table S1: Clinical scoring of domestic boars infected with ASFV “KAB6/2” and “SUM14/11”, Table S2: Clinical scoring of wild boar infected with ASFV “Germany 2020”, Table S3: Summary presentation of individual organ Cq values and genome copy numbers of ASFV “KAB6/2” and “SUM14/11” infected domestic boars, Table S4: Summary presentation of individual organ Cq values and genome copy numbers of ASFV “Germany 2020” infected wild boar.
